# An unusual presentation of pleomorphic adenoma in a patient with thalassemia: A case report

**DOI:** 10.1016/j.ijscr.2020.11.101

**Published:** 2020-11-21

**Authors:** Abdelrahman R. Alsaleh, Adham A. Aljariri, Baraa A. Wazwaz, Hasan A. Haider, Waheed Rahman, Abdulqadir J. Nashwan

**Affiliations:** a Otolaryngology Department, Ambulatory Care Center (ACC), Hamad Medical Corporation (HMC), Doha, Qatar; b Pathology Department, Hamad General Hospital (HGH), Hamad Medical Corporation (HMC), Doha, Qatar; c Hazm Mebaireek General Hospital (HMGH), Hamad Medical Corporation (HMC), Doha, Qatar; d University of Calgary in Qatar (UCQ), Doha, Qatar

**Keywords:** PA, Pleomorphic adenoma, ITF, Infratemporal fossa, WBC, White blood cells, AP, Anteroposterior, TR, Transverse, CC, Craniocaudal, H&E, Hematoxylin and eosin, MRI, Magnetic resonance imaging, Infratemporal fossa, Pleomorphic adenoma, Spontaneous infarction, Surgery, Parapharyngeal abscess, Thalassemia

## Abstract

We report the first case of spontaneous infarction occurring in pleomorphic adenoma located in infratemporal fossa mimicking parapharyngeal abscess, including clinical presentation, workups, and surgical approach. The final diagnosis was confirmed on a histological basis. The combination of CT scan and MRI might be helpful in distinguishing neoplasia from abscess collection in this area. Surgical intervention is the treatment of choice.

## Highlights

• Pleomorphic adenoma benign mixed tumor is a benign heterogeneous tumor.
• Infratemporal fossa infarcted neoplasms may present clinically as an abscess.
• The combination of CT scan and MRI might be a helpful diagnostic tool.
• Treatment of Infratemporal fossa infarcted neoplasms tumors is surgical.
• Transcervical approach allows excellent control of the tumor and neurovascular elements.

## 1 Introduction

Pleomorphic adenoma (PA) or benign mixed tumor is a benign heterogeneous tumor composed of variable epithelial and myeoepithelial components. PA is the most common salivary gland neoplasia that also can occur in the respiratory tract or nasal cavity [1].

Although infarction of PA after fine-needle aspiration has been documented thoroughly, unprovoked infarction of PA has remained as an unusual entity reported in the literature [2]. To our knowledge, our case report is the first case of spontaneous infarction occurring in pleomorphic adenoma located in the infratemporal fossa mimicking parapharyngeal abscess.

The infratemporal fossa (ITF) is located posterolaterally to the maxilla and maxillary antrum. It is bounded anteriorly by the maxilla and posteriorly by the glenoid fossa as well as the mandible. Medially, the ITF is bounded by the lateral pterygoid plates. The roof contains the foramen ovale and foramen spinosum and contains the pterygoid muscles.

Neoplasia involving the ITF region may arise from tissues in the region. However, more often, they result from the extension of the surrounding structures. Rarely, metastatic lesions can be seen in this region. Due to its concealed localization, tumors may remain unnoticed for quite some time; thus, signs and symptoms often appear late, insidious and may be falsely attributed to other disease processes [3].

## 2 Case presentation

A 20-year-old Mediterranean housewife female patient presented walk into the emergency department with a 5-day history of severe sore throat associated with difficulty of swallowing and trismus, which was not relieved with supportive treatments and oral antibiotics. Apart from minor thalassemia, her medical history was insignificant, and insignificant past surgical history; patient denied having fever, weight loss, night sweat, and loss of appetite, patient has no family history of malignancy, anyhow she reports multiple similar episodes of recurrent sore throat that responded to co-amoxiclav oral antibiotic.

**Fig. 1:**
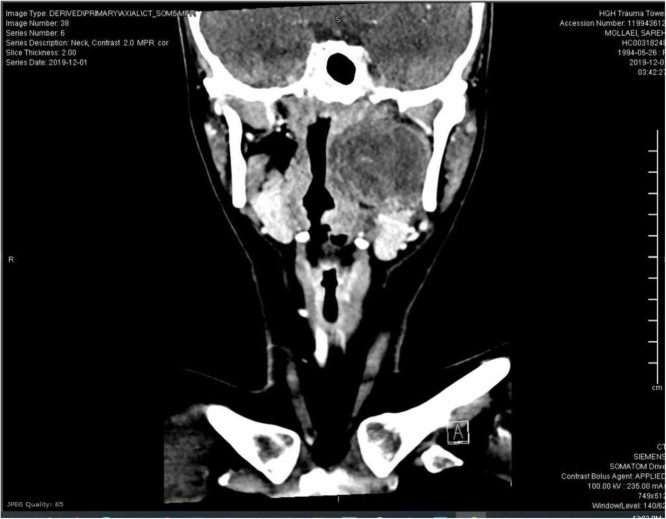
Coronal Cut of Neck CT with contrast.

Clinical examination revealed a left-sided peritonsillar swelling with a mild deviation of the uvula to the right side; she also had painful and tender submandibular lymph nodes on the left side as well. The fiberoptic exam showed mild medialization of the left parapharyngeal wall. Major salivary glands examination was unremarkable. Her routine blood tests were within normal limits except for the picture of microcytic hypovolemic anemia consistent with her thalassemia condition and elevated C-reactive protein (CRP) that was 28.9 (WBC count6.0 with no leukocytosis). Suspected to have a peritonsillar collection; aspiration was attempted in the emergency room that, however, yielded no pus. A computerized tomography (CT) neck with contrast (Fig. 1) was done to the patient and showed evidence of a large left parapharyngeal collection with enhancing peripheral wall and non-enhancing contents. The swelling measured 3.4 × 2.7 × 3.5 cm in the maximum anteroposterior (AP), transverse (TR) and craniocaudal (CC) dimensions, respectively. Multiple enlarged elongated benign looking submandibular and upper jugular groups of lymph nodes with preserved fatty hilum, largest measuring 9 mm in the short axis in the left submandibular region. Hence, the patient was presumed to have a left parapharyngeal collection - likely an abscess with enlarged lymph nodes.

The patient shifted to the operating theater for incision and drainage of left parapharyngeal abscess; trans-cervical approach was made initially by the ORL-HNS consultant to drain the suspected abscess. However, no pus yielded, repeated aspiration also did not yield anything. Therefore, a trans-oral approach followed where incision anterior to the anterior tonsillar pillar was done. With further dissection until reaching the left infratemporal fossa, the upper pole of a mass was noticed and was hard, surrounded by necrotic tissue. This full mass was delivered completely through transoral incision.

Post-operative, the patient tolerated the surgery very well and started diet short after the operation without any limitations; she was discharged on postoperative day 3 with analgesia.

The patient followed for 1 year and she was doing fine without any signs of recurrence, neurological deficit or palatal dysfunctional issues; no further imaging workup was done for the patient as the progression of the disease along with the final pathological report indicate a benign pathology.

**Fig. 2:**
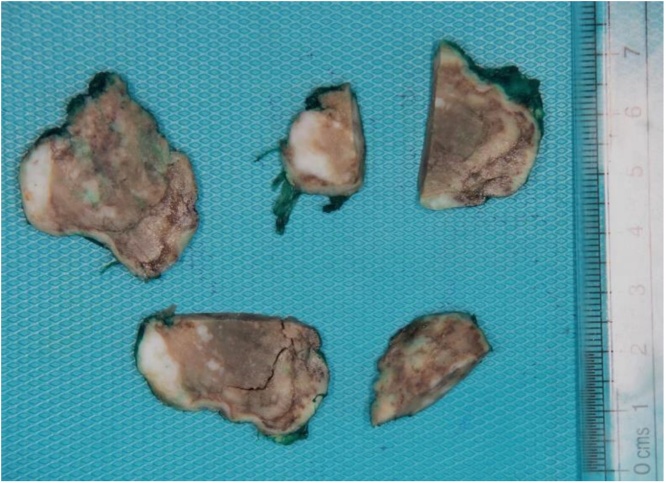
Representative sections of the tumor showing infarcted white parts, surgical margin inked green.

Histopathological; grossly, the specimen consisted of a single irregular firm tan brown soft tissue nodule measuring 4 × 3 × 2.5 cm. Cut surfaces showed brown heterogeneous soft tissue with multiple scattered white firm parts largest measured 1.5 × 1.5 × 0.5 cm abutting surgical margin (Fig. 2).

Fig. 2: Representative sections of the tumor showing infarcted white parts. Surgical margin inked green.

Microscopically, the extensively sampled specimen showed myoepithelial cells scattered within myxoid stroma intermingled with sheets of degenerate ductal epithelial cells together with prominent squamous metaplasia and abundant keratinization.

**Fig. 3:**
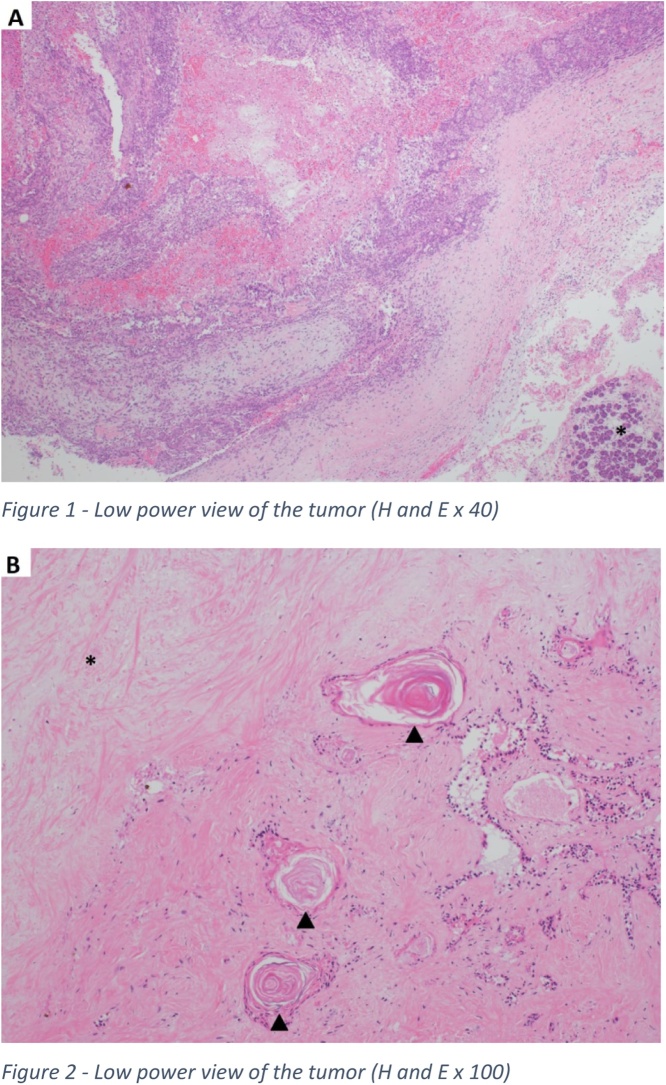
A: Tumor with a background of hemorrhage, inflammation, and a focus of normal salivary glands (asterisk) (H&E magnification x 40). B: infarcted part of the tumor (asterisk) with abundant squamous metaplasia and keratinization (arrow heads) (H&E magnification x 100).

Focal hemorrhage, inflammation, and necrosis were also noted. A rim of normal salivary gland tissue was identified. The overall appearances were consistent with an infarcted pleomorphic adenoma with florid squamous metaplasia (Fig. 3). No malignant features were identified.

Fig. 3 - Tumor with a background of hemorrhage, inflammation, and a focus of normal salivary glands (asterisk) (H&E magnification x 40).

## 3 Discussion

Tumors of the ITF present a diagnostic and a surgical challenge due to the often-occult nature of such lesions as well as the complexity of the local anatomy. Neoplasia of ITF can be classified as primary, secondary or metastatic [4]. Among the malignant tumors in the ITF region, adenoid cystic carcinoma, adenocarcinoma and squamous cell carcinoma are the most common malignant tumor encountered in this location [4]. In contrast, nasopharyngeal fibroma is frequently found in ITF benign lesions. To our knowledge, only three cases of pleomorphic adenoma in the ITF region were reported and we believe that we report the first case of an infarcted pleomorphic adenoma in the ITF region [[5], [6], [7]]. Several assumptions have been discussed regarding the origin of all these salivary gland tissue abnormal locations. Ferlito assumes the existence of scattered heterotopic salivary gland tissue in the head and neck region, and in the pituitary gland region specifically, external auditory meatus, nasal fossae, sternoclavicular joint, mandibula, and cervical soft tissues. Heterotopic salivary gland tissues in these areas, all have the potential to develop into a pleomorphic adenoma.

CT scan remains a substantial image modality in diagnosing tumors of the ITF as it helps to determine the extent of the disease, its local spread, and radiologic characteristics of the tumor may help to some extent in determining the type of the tumor.

Furthermore, Magnetic resonance imaging (MRI) should be considered as a modality whenever the suspicion of the vascular lesion is present or in cases of atypical presentation as in our case, the palate is the most common site of minor salivary gland PA followed by the lip, buccal mucosa and floor of the mouth, tongue, tonsil pharynx, retromandibular area and nasal cavity, parapharyngeal space PA can originate de novo or from the deep lobe of the parotid gland and then extend by the stylomandibular tunnel into the parapharyngeal space.

Parapharyngeal tumors are rare, constituting less than 0.5% of head and neck neoplasms [8]. Of these, PA is the most common benign tumor, representing around 40% [9].

Due to its concealed location, lesions often appear late; in addition, surgical planning is confounded by proximity to vital structures, namely intracranial structures, the orbit, sinuses, and the nasopharynx. Indicating the difficulty of access, numerous surgical approaches have been described.

Although the prognosis of PA is generally good, yet the conventional watchful waiting and clinical follow up is not sufficient, while periodic radiological evaluation might be needed to detect any recurrence post-surgical excision.

While many surgeons acclaim complete surgical excision is the only valid treatment option for pleomorphic adenoma, other reports illustrated good results with adjuvant radiotherapy against incompletely resected tumors due to its location that renders them inoperable, However, this remains a matter of controversy [2].

Although seems to be relatively rare, spontaneous infarction resembling abscess formation should be taken into consideration in the differential diagnosis of such cases. In atypical case presentations, one might consider MRI to differentiate between abscess and neoplasia in the ITF region. This case has been reported in line with the SCARE Guideline 2018 [10].

## 4 Conclusion

ITF Infarcted neoplasms may present clinically as an abscess, in these situations, the combination of CT scan and MRI might be a helpful diagnostic tool in distinguishing neoplasia from abscess collection in this area. Treatment of ITF tumors is surgical. The transcervical approach allows excellent control of the tumor and neurovascular elements but the transoral approach might be also needed according to the case nature.

## Declaration of Competing Interest

No conflict of interest.

## Funding

Non sponsored and no fund received.

## Ethical approval

The paper describes a case report therefore no special ethical consideration required.

## Consent

Written informed consent was obtained from the patient for publication of this case report and accompanying images. A copy of the written consent is available for review by the Editor-in-Chief of this journal on request.

## Author contribution

ARA: Data Collection, Literature Search, Manuscript Preparation,

AAA: Manuscript Preparation & revision,

HAH, WR: Manuscript Preparation,

BAW: pathology slides preparation,

AJN: Manuscript Revision and submission.

All authors read the final manuscript and agreed on it.

## Registration of research studies

N/A.

## Guarantor

Dr Abdelrahman R. Alsaleh

AAlsaleh@hamad.qa

00974-55176528

## Provenance and peer review

Not commissioned, externally peer-reviewed.

## References

[1] Behzatoglu K. (2005). Spontaneous infarction of a pleomorphic adenoma in parotid gland: diagnostic problems and review. Diagn. Cytopathol..

[2] Clark M. (1999). CT of intranasal pleomorphic adenoma. Neuroradiology.

[3] Tiwari R. (2000). Tumors of the infratemporal fossa. Skull Base Surg..

[4] Conley J.J. (1964). Tumors of the infratemporal fossa. Arch. Otolaryngol..

[5] El-Hadi T. (2009). Plemorphic adenoma of the infratemporal space: a new case report. Int. J. Otolaryngol..

[6] Gurey L.E., Brook C.D., Parnes S.M. (2010). Pleomorphic adenoma of the infratemporal fossa: case report and literature review. Laryngoscope.

[7] Jeyanthi K. (2007). Pleomorphic adenoma in the infra-temporal space: the first case report. Head Neck Pathol..

[8] Batsakis J.G., Sneige N. (1989). Parapharyngeal and retropharyngeal space diseases. Ann. Otol. Rhinol. Laryngol..

[9] Hughes K.V., Olsen K.D., McCaffrey T.V. (1995). Parapharyngeal space neoplasms. Head Neck.

[10] Agha R.A., Borrelli M.R., Farwana R., Koshy K., Fowler A., Orgill D.P., For the SCARE Group (2018). The SCARE 2018 Statement: updating consensus surgical CAse REport (SCARE) guidelines. Int. J. Surg..

